# Systematic Review and Meta-Analysis of 12 Randomized Controlled Trials Evaluating the Efficacy of Invasive Radiofrequency Treatment for Knee Pain and Function

**DOI:** 10.1155/2019/9037510

**Published:** 2019-06-26

**Authors:** Tao Hong, Haiyuan Wang, Guangxiao Li, Peng Yao, Yuanyuan Ding

**Affiliations:** ^1^Department of Pain Management Center, Shengjing Hospital of China Medical School, No. 36, Sanhao Street, Heping District, Shenyang 11004, China; ^2^Department of Intensive Care Medicine, Shengjing Hospital of China Medical School, No. 36, Sanhao Street, Heping District, Shenyang 11004, China; ^3^Medical Record Management Center, The First Affiliated Hospital of China Medical University, No. 155, Nanjing Bei Street, Shenyang 110001, China

## Abstract

Radiofrequency (RF) treatment is an invasive and promising procedure in the treatment of osteoarthritis (OA). A meta-analysis based on 12 RCT studies was to investigate whether invasive RF treatment is more effective in relieving knee pain and improving knee function. Relevant studies were searched on database of Pubmed, Embase, EBSCO, Cochrane library, Wanfang digital database, VIP database, and CNKI up to January 2018. A total of 841 participants from 12 publications were included. The weighted mean difference (WMD) and the corresponding 95% CIs were used to evaluate the difference in pain scores and OKS/WOMAC scores between RF treatment and control groups. The statistical analysis was performed by Stata 12.0. The pain scores (VAS) in the RF group were lower than those in the conservative treatment group after 1 week (WMD -1.77, 95% CI -2.93 to -0.61, P<0.01), 1 month (WMD -1.40, 95% CI -1.98 to -0.82, P<0.01), and 3 months (WMD -1.32, 95% CI -2.27 to -0.37, P<0.01) of treatment, while there was no significant improvement in knee function. In subgroup analyses by site of radiofrequency, RF mode showed some discrepancies in the WMD of VAS between the treatment and control groups. In addition, subgroup analysis and meta-regression showed that the efficacy of RF treatment for reducing pain is reversely related to female ratio, and we did not find any surgery-related adverse reactions. RF treatment significantly reduces the knee pain, but rarely improves the knee joint function. Radiofrequency ablation has better efficacy than pulsed radiofrequency ablation in reducing pain. Furthermore, subgroup analysis and meta-regression suggested that women are more sensitive to RF treatment than men.

## 1. Introduction

Osteoarthritis (OA) is a common chronic noninflammatory joint disease characterized by articular cartilage damage, which involves the subchondral bone, synovium, and periarticular tissues. The probability of suffering from symptomatic knee OA in elderly patients aged >65 years is 20–30% [1]. Currently, the disease-modifying treatment for OA is lacking. The therapeutic methods are limited to controlling pain, delaying cartilage destruction, and improving mobility and quality of life [2]. The conservative treatments include weight loss, physical therapy, oral nonsteroidal anti-inflammatory drugs (NSAIDs), or intra-articular injections of hyaluronic acid and corticosteroid. The elderly patients, who have failed the above conservative treatment might prefer arthroscopic surgery or total knee arthroplasty (TKA) [3]. However, about 20–53% of the patients continue to suffer from persistent and severe pain around knee area after TKA [4–6].

Invasive radiofrequency treatment can fill the gap between conservative treatment and TKA. It is a promising procedure with advantages of minimal invasion, rapid recovery, and few adverse events, including radiofrequency ablation (RFA) on the genicular nerve, intra-articular pulsed radiofrequency ablation (PRF), and cooled radiofrequency ablation (CRF). It can be applied to patients with severe complications, who cannot undergo TKA, those with severe pain around the knee joint area after TKA, those with severe joint pain who refuse to undergo surgery, and those who have failed oral NSAIDs or knee intra-articular injection.

The exploration of RFA on the articular nerve was first proposed by Choi et al. [7]. Their target nerves were superior lateral (SLGN), superior medial (SMGN), and inferior medial (IMGN) [7, 8], and others also selected medial retinacular nerve and the infrapatellar branch of the saphenous nerve as the target points [9]. The electrode heat of RFA is ranging from 70°C to 80°C for 90-180 seconds. The patients experienced significant pain relief and functional improvement [7–15]. 50% pain relief rate at 3 and 5 months is 59% and 62.5%. When the intra-articular PRF is applied, the radiofrequency needle should be placed in the joint cavity, and the tip of needle's temperature is no more than 42°C for 5-15 min. 50% remission rate at 6 months is 35.5-100% [16–21]. CRF was first introduced in 2015, in which temperature at the tip of the needle was regulated around 60°C by the water circulation. This new technology created a greater local neuron denervation than the traditional RFA, and 50% pain relief rate at 6 months is 74.1% [6, 22–28]. Based on the above literatures, we can conclude that knee-related RF treatments can effectively control knee pain and improve knee function. The three RF treatments modes appear to be safe and effective treatments, and there are no reports of serious complications. The reviews published previously tend to describe the target point of treatment, post-procedure pain relief rates, adverse reactions, and flaws and limitations of each literature [29, 30]. Their included articles are mainly retrospective study or case series. The conclusions of these reviews require reading carefully, because their included articles are few RCTs, small surgical sample size, and low quality. In addition, different RF treatment methods still lack quantitative analysis of the degree of knee pain and functional relief. Therefore, we searched on the English and Chinese databases, included RCTs for different invasive RF treatments and then extracted the objective data (NRS/VAS, OKS/WOMAC) to quantitatively compare the long-term analgesic effects and knee function improvement of three RF treatment methods, thereby providing objective guidance to clinicians in selecting an optimal OA treatment method.

## 2. Materials and Methods

### 2.1. Data Sources, Search Strategy, and Study Selection

The electronic databases, Pubmed, Embase, EBSCO, and Cochrane Central of Controlled Trials (Central), as well as Wanfang digital database, VIP database, and CNKI (Chinese database), were searched to identify eligible studies since inception to January 2018. An in-depth literature search was performed using the following keywords: “knee pain” or “chronic pain” or “radiofrequency” or “genicular nerve” or “nerve ablation” or “intra-articular” or “thermal radiofrequency” or “knee osteoarthritis”. Each search was limited to human clinical trials in English and Chinese languages. Initially, the studies were screened according to the title, abstract, and keywords to exclude the irrelevant studies. Then, the full text of the enrolled and potentially eligible studies was carefully screened to further identify the eligible studies. In addition, the reference lists of the retrieved studies were manually checked to find out relevant studies.

#### 2.1.1. Inclusion Criteria

(1) RCTs on clinical efficacy of invasive radiofrequency treatment for OA were performed; (2) patients in the experimental group received any of the following three modes of radiofrequency: CRF, RFA, and PRF; (3) a control group was involved; (4) the visual analogue scale/numerical rating scale (VAS/NRS) or Oxford Knee Score/the Western Ontario and McMaster (OKS/WOMAC) at different time points post-treatment were provided, with their mean and standard deviations (SD) or the standard error (SE) available to calculating the weighted mean differences (WMD) and the corresponding 95% confidence intervals (CIs).

#### 2.1.2. Exclusion Criteria

(1) Patients who had underwent knee arthroplasty and arthroscopic surgery will be excluded, though they might also suffer from serious knee pain; (2) when there were duplicate studies, only the newly published or largest sample size ones were included; (3) studies with unextractable or insufficient data were also excluded; (4) non-RCT studies were excluded; (5) unpublished studies such as conference abstracts were excluded because of their poor quality and insufficient data.

### 2.2. Data Collection

Two experienced researchers used a predesigned form to independently extract data. Any uncertainty or discrepancy was resolved through discussion. The following parameters were included: name of the first author, year of publication, country of publication, location of study population, study design, Kellgren–Lawrence classification, mode of RF, operating site of RF, sample size of the experimental and control groups, age, gender, VAS/NRS, and OKS/WOMAC score at different time points before and after treatment. Since VAS and NRS are comparable to some extent, we converted NRS to VAS score in the subsequent data extraction for uniform statistical analysis.

### 2.3. Quality Assessment

**Figure 1:**
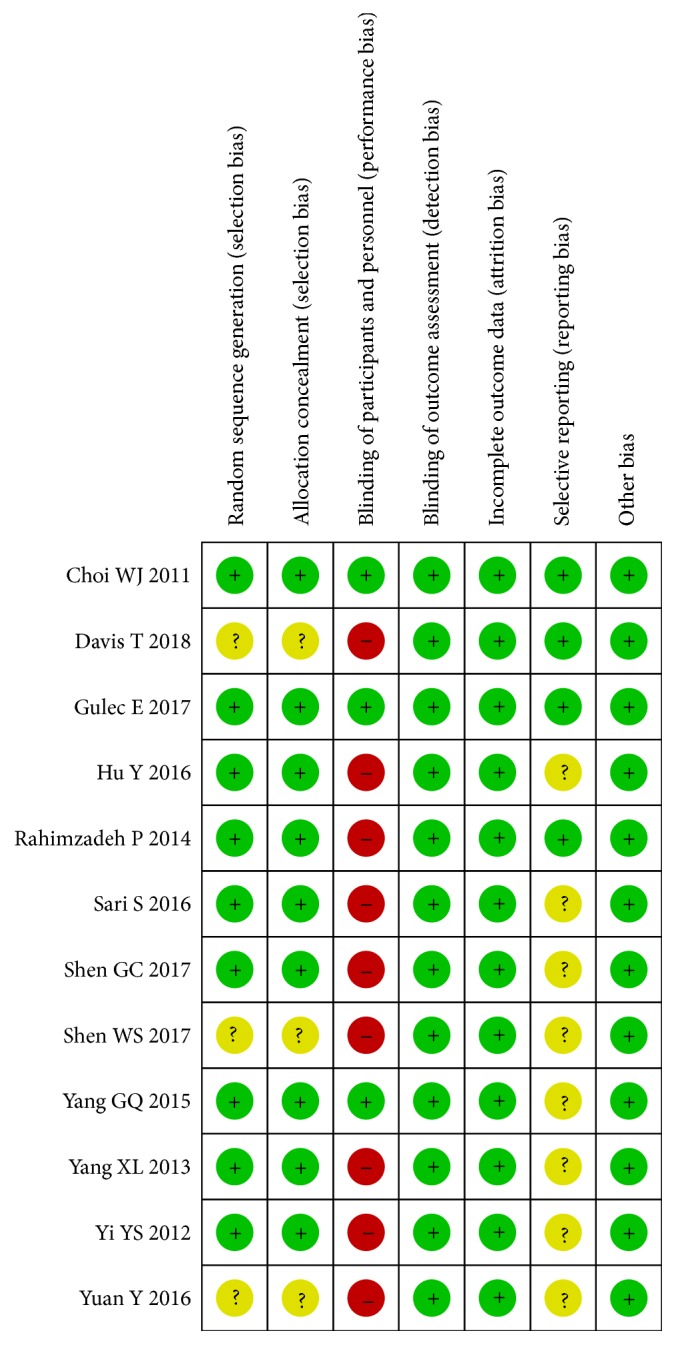
Risk of bias summary: review authors' judgements about each risk of bias item for each included study.

**Figure 2:**
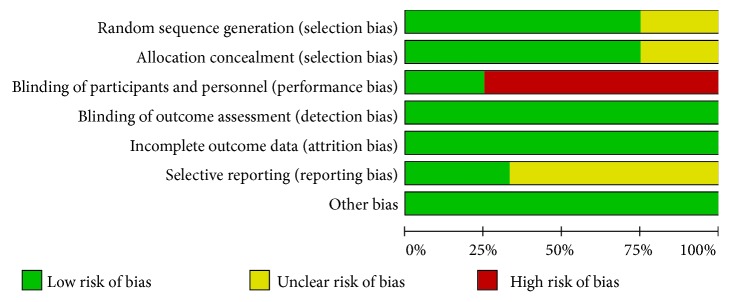
Risk of bias graph: review authors' judgements about each risk of bias item presented as percentages across all included studies.

Risk of bias for each study was independently assessed by two researchers (HT and GXL) using the criteria outlined in the Cochrane Handbook for Systematic Reviews of Interventions [31]. Disagreements would be resolved by discussing or consulting with another author to reach consensus. RCTs were evaluated in terms of allocation sequence generation (selection bias), concealment of allocation (selection bias), outcome assessment (performance bias), incomplete outcome data (attrition bias), selective outcome reporting (reporting data), and other bias. We graded risk for each potential source of bias as high, low, or unclear and conducted justification for judgements in the ‘Risk of bias' table (Figures 1 and 2).

### 2.4. Statistical Analysis

The WMDs and the corresponding 95% CIs were used to evaluate the difference in pain scores and OKS/WOMAC scores between the RF treatment and control groups at different time points (1 week, 1 month, and 3 months after treatment). Heterogeneity between studies was evaluated using the Cochran Q test and* I*^2^statistics,* P* < 0.05 or* I*^2^> 50% indicated that the heterogeneity between studies was statistically significant [32]; then the random-effects model was used to calculate the poor WMD; otherwise, fixed-effect model would be used. To explore the sources of heterogeneity, subgroup analyses were conducted based on variables such as location, concealment of location, RF modes, concomitant therapy, site of RF, and sex ratio in cases (female versus male). To further clarify the source of heterogeneity, metaregression analyses were performed based on different subgroup analysis variables [33]. To observe the impact of any single study on the pooled WMD, sensitivity analyses were conducted by sequentially eliminating individual study one by one following by a recalculation of the WMD and the corresponding 95% CIs. Publication bias was examined by using the adjusted rank correlation test and the regression asymmetry test by Begg and Egger et al. [34, 35]. Due to the limited number of studies on the OKS/WOMAC scores, subgroup analysis, meta-regression analysis, and publication bias analysis for the WMDs were not performed. All analyses were calculated using Stata 12.0 (Stata Corporation, College Station, TX).

## 3. Result

### 3.1. Study Characteristics

**Figure 3:**
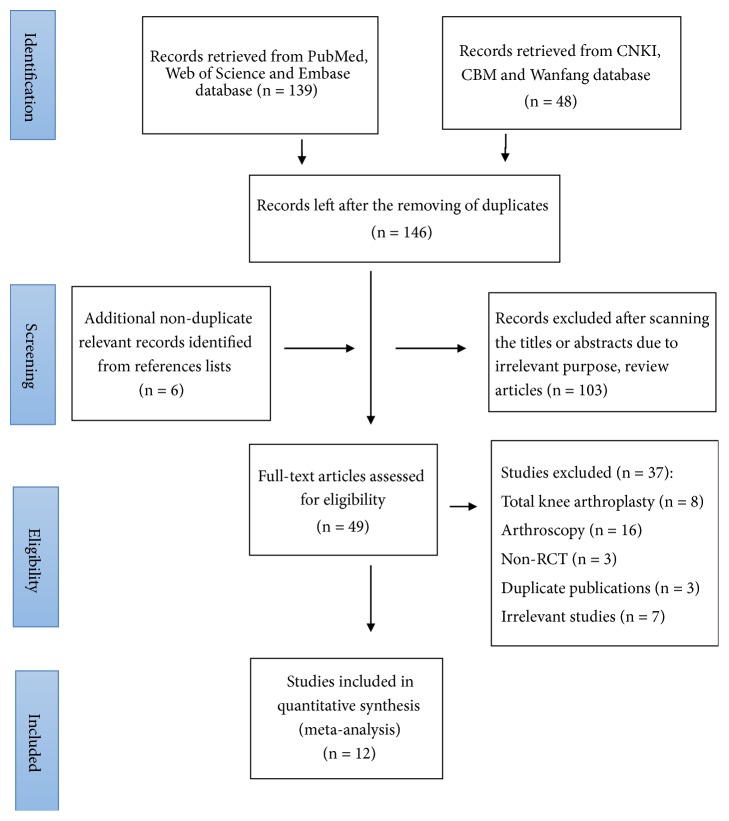
Flow diagram showing the process for study selection.

According to our searching strategy, 187 studies were retrieved. Finally, 12 RCTs fulfilled the inclusion criteria, including 9 studies from Asia, 2 from Europe, and 1 from the USA (Figure 3). Among these studies, the one by Rahimzadeh et al. [18] adopted two different control treatments. Then it was divided into two substudies. Hence, a total of 13 substudies were included in the present meta-analysis. Of them, VAS/NRS scores were available in 13 substudies for the comparison of pain improvement between the RF treatment and the control groups at different time points before and after treatment. In addition, WOMAC and OKS scores were available in 3 studies, respectively, to evaluate the knee function improvement between the RF treatment and control groups at different time points. Detailed information of studies included is illustrated in Table 1. The intervention parameters, results, adverse reactions, and limitations of studies included are listed in Table 2. Since the original paper does not provide statistical power and minimal clinically important difference (MCID), this part of the information cannot be extracted.

### 3.2. Risk of Bias of the Eligible Studies

#### 3.2.1. Allocation

David et al. [26], Shen et al. [10], and Yuan et al. [16] did not mention the specific allocation method. Other investigators used methods as “computer-generated list” or “computer-generated schedule”. Therefore, we assessed them at low risk of bias.

#### 3.2.2. Blinding

Three studies by Choi et al. [7], Gulec et al. [17], and Rahimzadeh et al. [18] had a double-blind design, in which the surgeons and assessor staff were unaware of allocation. Thus, the detection and performance bias were considered to be of low risk. For the studies in which blindness was not mentioned, the detection bias and performance bias were treated as low risk and high risk, respectively.

#### 3.2.3. Incomplete Outcome Data

In Choi et al. [7] and David et al. [26], more than 90% (126/138, 35/38) of participants complete follow-up. We assessed them as having low risk. There were no dropout in other studies, and they were at low risk.

#### 3.2.4. Selective Reporting

Choi et al. [7], David et al. [26], Gulec et al. [17], and Rahimzadeh et al. [18] provided their trial register number, and we assessed them as having a low risk. Other studies are at unclear risk.

#### 3.2.5. Other Potential Sources of Bias

We identified no other potential sources of bias.

### 3.3. Main Result

**Figure 4:**
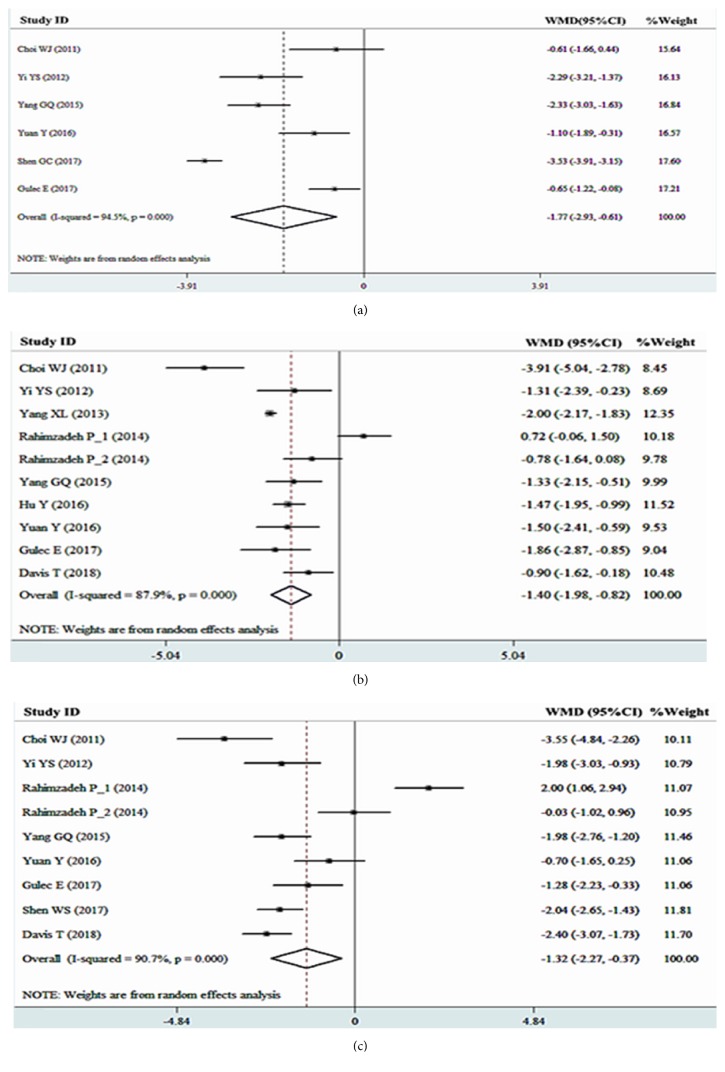
Forest plots for the associations between radiofrequency treatment and VAS scores (a) at 1 week, (b) 1 month, and (c) 3 months.

Because the between-study heterogeneity was statically significant (1 week, ***I***^***2***^=94.5%, ***P***<0.01; 1 month, ***I***^***2***^=87.9%, ***P***<0.01; 3 months, ***I***^***2***^=90.7%, ***P***<0.01), the random-effects model was used to calculate WMDs and the corresponding 95% CIs. The results showed that the pain scores of the RF treatment group were lower than those of the control group at 1 week, 1 month, and 3 months after treatment [1 week, WMD -1.77, 95% CI -2.93 to -0.61, P<0.01 (Figure 4(a)); 1 month, WMD -1.40, 95% CI -1.98 to -0.82, P<0.01(Figure 4(b)); 3 months, WMD -1.32, 95% CI -2.27 to -0.37, P<0.01 (Figure 4(c))].

Similarly, due to the limited number of included studies and a relatively large between-study heterogeneity for OKS/WOMAC scores, the random-effects model was used to merge the effect sizes except that the fixed-effects model was used for OKS score at 1 week after treatment. The majority of the results showed that RF treatment did not improve the knee function significantly (Figures S1 and S2). OKS [1 week, WMD -3.16, 95% CI -5.6 to -0.72, P=0.01 (Figure S1a); 1 month, WMD -4.11, 95% CI -12.41 to 4.19, P=0.33(Figure S1b); 3 months, WMD -1.59, 95% CI -13.34 to 10.17, P=0.79 (Figure S1c)]; WOMAC [1 week, WMD -3.66, 95% CI -13.12 to 5.80, P=0.45 (Figure S2a); 1 month, WMD -7.58, 95% CI -16.22 to 1.05, P=0.09(Figure S 2b); 3 months, WMD -1.25, 95% CI -7.33 to 4.84, P=0.69 (Figure S 2c)]

### 3.4. Subgroup Analysis

As illustrated in Table 3, the WMDs of pain scores between the RF group and control groups at different time points after treatment were subjected to subgroup analyses based on the location, blindness, RF mode, concomitant therapy (except for RF treatment, intra-articular serum injection or pain point blockage or intra-articular hyaluronic acid injection was applied), site of radiofrequency, and sex ratio.

Subgroup analysis showed that RFA of the genicular nerve could significantly relieve pain from 1 week to 3 months after treatment (1 week, WMD -1.80, 95% CI -2.81 to -0.78, P<0.01; 1 mon, WMD -1.83, 95% CI -2.57 to -1.09, P<0.01; 3 months, WMD -2.36, 95% CI -2.92 to -1.80, P<0.01), while the intra-articular PRF was slow-acting (1 week, WMD -1.77, 95% CI -3.82 to 0.27, P=0.09), which could relieve the pain only at 1 month (1 month, WMD -0.97, 95% CI -1.86 to -0.08, P=0.03), but showed an insignificant improvement at 3 months (3 months, WMD -0.43, 95% CI -1.84 to 0.98, P=0.55).

The RF mode was also one of the reasons for the improvement in pain. CRF (no data in 1 week) and RFA treatment showed a significant improvement in pain at all observation time points. However, PRF treatment did not show any statistically significant improvement in the pain till the 3^rd^ month (WMD -0.00, 95% CI -1.42 to -1.42, P=0.99). Subgroup analysis by sex ratio showed a significant difference in pain scores between the treatment and control groups. At 1 week and 3 months, the WMD of the pain score was merely statistically significant when the sex ratio was ≥ 2 (1 week, WMD -1.92, 95% CI -3.42 to -0.43, P=0.01; 3 months, WMD -2.00, 95% CI -2.97 to -1.04, P<0.01). Conversely, WMD was not statistically significant when the sex ratio was < 2 (1 week, WMD -1.48, 95% CI -3.12 to 0.17, P=0.08; 3 months, WMD -0.76, 95% CI -2.30 to 0.78, P=0.34). In addition, the application of blindness and the presence or absence of concomitant therapy exerted a specific influence on the results.

### 3.5. Meta-Regression Analysis

**Figure 5:**
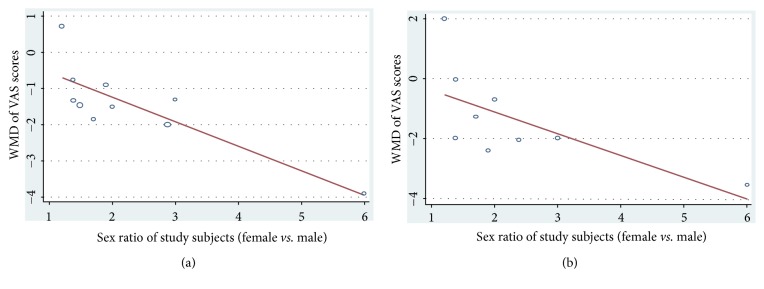
Meta-regression reveals inverse correlations between sex ratio and the weighted mean difference (WMD) of VAS scores at (a) 1 month and (b) 3 months.

Due to the significant heterogeneity among the study groups and limited findings from subgroup analyses, meta-regression analyses were conducted to further verify the source of between-study heterogeneity. At 1 and 3 months after treatment, the sex ratio was inversely correlated to the WMD of VAS. The slope of the meta-regression curve at 1 month was -0.67, 95% CI -1.08 to-0.27, P<0.01, which might explain 71.56% heterogeneity across studies, while that at 3 months was -0.73, 95% CI -1.48 to 0.03, P=0.06, which might account for about 36.88% heterogeneity across studies (Figures 5(a) and 5(b)).

### 3.6. Sensitivity Analysis

Sensitivity analysis was used to evaluate the stability robust of the pooled WMDs. After the sequential exclusion of individual studies one by one, the WMDs were recalculated to identify the significant change in our results. Sensitivity analysis the elimination of any single study was unlikely to overturn our findings (Figure S3a-c).

### 3.7. Publication Bias

Evaluation using Begg's correlation tests (1 week: ***P***=0.45; 1 month, ***P***=0.28; 3 months, ***P***=0.75) or Egger's regression test (1 week: ***P***=0.15; 1 months, ***P***=0.19; 3 months, ***P***=0.49) did not show any publication bias in this meta-analysis.

## 4. Discussion

In the present study, 12 RCTs involving a total of 841 participants were enrolled to evaluate the efficiency of RF treatment on long-term analgesia and improvement in the knee function in OA. Our results showed that the pain scores in the treatment group were lower than those in the control group at 1 week, 1 month, and 3 months after RF, while there was no significant improvement in knee function. In the subgroup analysis, CRF (no data in 1 week) or RFA in the genicular nerves significantly improved the pain within 1 week, and the analgesic effect could maintain up to 3 months. Nevertheless, intra-articular PRF was slow-acting with a short duration, its pain relief effects appeared until 1 month, but vanished at 3 months. Thus, it is suggested that RF thermocoagulation of genicular nerves can improve the refractory pain of the knee joint and the quality of life of patients. In addition, the pain relief effects of RF treatment were inversely associated with sex ratio. The higher the female ratio was, the more effective the pain relief effects were. We did not find any surgery-related adverse reactions. Furthermore, no publication bias was found in the current study, and the sensitivity analysis suggested that the pooled effect sizes did not alter significantly after the exclusion of individual studies sequentially, which confirmed the stability of the results.

The mechanism of intra-articular PRF or RFA of genicular nerves has not yet been clarified; however, all published studies suggest that RF treatment can significantly reduce pain around the knee [7, 10–14, 16–19, 21, 26–28, 36–40]. The analgesic effect of PRF at 1 week of treatment was not statistically significant and putatively associated with a large space of knee joint cavity and a large distance of the needle cannula from the peripheral sensory nerve around the synovium, cartilage, and bone [17, 38]. Sluijter et al. [21] speculated that the electric field generated by PRF could play a therapeutic role by affecting the immune cells to produce proinflammatory cell mediators or inhibit the excitation of C-fibers and synaptic transmission. Moffett et al. [39] postulated that the increased secretion of endogenous opioid precursor mRNA and corresponding opioid peptides in human dermal fibroblasts and human epidermal keratinocytes after PRF might be one of the mechanisms of analgesia. After 5 and 6 months, the rates of intra-articular PRF pain remission in the knee joint cavity were 50% and 35.5%, respectively [38, 40]. However, the RFA on the genicular nerves block the nociceptive pain (A-б and C-fibers) from transmitting to the central nervous system without destroying the motor or sensory nerves (A-*β* fiber). During RFA, the high temperature of the electrode tip of the RFA disrupts the nerve tissue with respect to protein denaturation and coagulation necrosis, which can significantly reduce the rest pain but have a limited effect on the activity pain [10]. The efficacy of RFA in the nerves around the knee joint can last for 6 months or 1 year, when 64% and 32% patients, respectively, continue to present pain relief rate of > 50% [26]. Compared to traditional RFA, the CRF changes the shape and radiation range of the electric field generated by the needle tip. The current is >10-fold that of radiofrequency thermocoagulation. However, the water circulation inside the needle tip can maintain the tip temperature at 60°C, which enables expanding the denervated area and reduce the surgical failure rate. In the randomized prospective multicenter trial conducted by Davis et al. [26], the proportion of patients with > 50% pain remission rate was 74% and 16% in CRF and control groups, respectively, after 6 months of observation. McCormick et al. [27, 28] reported that the proportion of patients with > 50% pain remission rate ranging from 35 to 74.1% at 6 months. Furthermore, comparing the scores for pain remission, RFA or CRF of the genicular nerves was more effective than PRF for analgesia in the knee joint at the 6^th^ month, which was similar to the results of the subgroup analysis in the current study. However, most of the studies mentioned above were retrospective analyses and lacked a control group, which did not allow the comparison of the curative effect with the traditional treatment. Moreover, only one RCT was related to CRF in the genicular nerves. Thus, additional clinical trials are essential for exploring the efficiency of analgesia.

In this meta-analysis, among the 7 studies on RCT of RFA in the genicular nerves, only 2 studies had performed prognostic nerve blocks with local anesthetic, in which patients with a remission rate of > 50% were enrolled in the RFA group, and 12–25.9% showed ineffective treatment or did not respond to the treatment [7, 37]. 1-2 mL lidocaine is often used for preoperative nerve block around pain generator, as it can block the target nerves as well as the surrounding sensory nerves. However, RF is unable to encompass the whole area. In addition, the anatomical variation or the operator's experience and operation methods would hinder the effect of the blockade. Consequently, ultrasonographic guidance for finding the nerves would significantly improve the effect of the blockade or RF treatment. McCormick et al. speculated that > 80% remission rate of the diagnostic obstruction of the genicular nerves was positively correlated to the CRF success rate [28].

Based on the published studies, the three RF methods can improve the knee function [7, 10, 12, 13, 16–19, 26, 37]. PRF can improve the knee function by regulating the release of immune inflammatory mediators and interfering with the inflammatory pathways related to the occurrence and development of OA. Vas et al. [41]. compared the knee DR before and after PRF on the genicular nerves and found that the patellofemoral joint and tibiofemoral joint spaces were widened as compared to those before the procedure, which was supposedly associated with a decline in the pain-induced muscle spasm or stiffness. However, the current results suggested that the RF treatment did not only revealed any significant difference in the knee function improvement, while the OKS score showed a difference at 1 week, which was inconsistent with the findings in the literature. Since WOMAC or OKS was adopted in 6 of the enrolled studies, the accuracy of the results necessitates further verification.

No surgical-related adverse reactions were reported during RF treatment group. Some studies stated severe pain during surgery, which might be attributed to the electrode tip of the needle that was close to the periosteum or tendon and could be reduced by replacing the needle site [42]. Some studies mentioned that preoperative or intraoperative use of analgesic and sedative drugs could reduce the rate of pain; however, osteonecrosis might occur after burning the periosteum at 60°C if it was not be found promptly [43]. Local subcutaneous bleeding was commonly observed at the puncture site; however, it was not statistically significant as compared to the control group due to the absence of local hematoma. 77% of the RF thermocoagulation group experienced prolonged hypoesthesia, which gradually shrank or disappeared after 2–6 weeks [9].

Subgroup analysis showed the sex ratio indicators of better pain improvement. The meta-regression analysis further proved that the higher the female proportion, the better the treatment effect, although the underlying mechanism is yet to be elucidated. Women are prone to suffering from chronic pain syndrome, and the frequency, areas of the body, duration, and severity of pain are significantly higher than those in men [44–46]. In addition, women are rather willing to report the pain situation to the doctor, which might also be one of the causes of the difference in pain between the genders [47]. OA primarily occurs in elderly women; the changes in the gonadal hormones in the body comprise the factors that affect women's perception and sensitivity to pain. For example, postmenopausal patients may experience transient joint pain after estrogen replacement therapy [48], and the pain score during the menstrual cycle phase will increase significantly [49]. The levels of estrogen, progesterone, and androgen can regulate pain sensitivity, which might be related to the endogenous opioid system [50].

For all the RCTs included in our studies published in the past 9 years, their limitations are summarized as follows. (1) Some of the researches were conducted based on relatively small sample size (35 to 42 patients) [7, 14, 16]. (2) The body mass index (BMI) of patients was not mentioned in most literatures, except for three studies [11, 14, 16]. Established evidence showed strong correlation between obesity and the incidence of OA. It was reported that the probability of developing OA of the knee was decreased by 50% with a 10-pound weight loss over 10 years [51]. (3) Shen et al. [10] did not provide the technical details of the procedure. (4) The observation end points were 2 months [36], 3 months [7, 10, 11, 13, 14, 17, 18], and 6 months [12, 16, 19, 26], respectively, lacking long-term clinical observation. (5) Different outcome measurements were used to evaluate knee function changes in the literatures, including WOMAC, OKS, Lysholm, ROM, and AKSS. WOMAC [11, 16, 17, 19] and OKS [7, 13, 26] were most commonly used to indicate knee motion improvement after RF treatment. (6) Shen et al. [10] and Yang et al. [36] suspend analgesic drugs to observe the therapeutic effect after procedure; Hu et al. [19] and Sari et al. [11] did not impose additional drugs or physiotherapy on OA patients after procedure, but did not clearly mention pre- and postprocedure medication; the rest of the literatures did not record any analgesic consumption, which may affect the evaluation of postprocedure efficiency. Readers should be cautious in drawing conclusions from this review because of the limitations mentioned above.

In the present study, we conduct a pooled analysis of improvement in the pain and knee joint function with RF treatment at different time point using meta-analysis method. The absence of published bias in the enrolled studies and sensitivity analysis indicated the robustness of the results. Nevertheless, the present study has certain limitations. Firstly, the WMDs of all combined trials showed a statistically significant reduction in VAS scores at some time points, but this difference was below the widely accepted, minimally clinically significant decrease in VAS of at least 2/10. Second, the blind method was adopted in only 3 studies, indicating a potential high risk of bias. This might be because blindness was not applicable in some studies. Third, Few or no data was related to cartilage mechanism or change in morphology. In addition, only one RCT of CRF was included in our analysis. The comparability of this treatment with other types of RF treatment needs further investigation.

## 5. Conclusion

In summary, our meta-analysis showed that, in comparison with the conservative treatment, invasive RF treatment significantly improved the knee joint pain, but exerted a limited effect in improving the knee joint function. In addition, CRF or RFA on genicular nerves exhibited a more significant and long-lasting analgesic effect as compared to intra-articular PRF. Furthermore, women are more likely to benefit from RFA treatment on the genicular nerves around the knee joint than men. Both subgroup analysis and meta-regression suggested a positive correlation between knee pain remission and female in the gender ratio, although the mechanism is not yet clarified. Due to the limitations of included literatures, the authenticity of the results requires further verification by prospective multicenter randomized controlled trials.

## Figures and Tables

**Table 1:** Basic information of the 12 observational studies included in the current meta-analysis.

First author	country	location	Sample size	Treatment	Control	Kellgren-Lawrence grade	Treatment VAS baseline	Control VAS baseline
(year)				M/F	M/F			
Choi WJ (2011)[7]	Korea	Asia	35	2/15	3/15	2-4	7.82 (1.38)	7.72 (0.75)
Shen WS (2017)[10]	China	Asia	54	7/20	9/18	NA	7.12 (1.08)	7.14 (1.03)
Yuan Y (2016)[16]	China	Asia	42	7/15	7/13	NA	5.9 (1.1)	5.6(1.4)
Gulec E (2017)[17]	Turkey	Europe	100	17/33	20/30	2-3	7.96 (1.78)	5.5 (1.48)
Rahimzadeh P(2014)[18]	Iran	Asia	70	11/13	9/11 10/16	1-3	7.08 (1.41)	6.65(0.98) 7.11 (1.03)
Sari S (2016)[11]	Turkey	Europe	73	7/30	9/25	2-4	NA	NA
Davis T (2018)[26]	US	America	151	26/50	26/49	2-4	7.2 (1.2)	6.9 (1.4)
Shen GC (2017)[12]	China	Asia	60	6/24	5/25	3-4	5.51(1.32)	5.79(0.88)
Yang GQ (2015)[13]	China	Asia	62	14/17	12/19	3-4	7.25 (1.33)	7.21 (1.58)
Yi YS (2012)[14]	China	Asia	36	NA	NA	NA	7.26 (1.34)	7.19 (1.57)
Hu Y (2016)[19]	China	Asia	92	17/28	20/27	2-3	6.53 (1.1)	6.38 (1.03)
Yang XL (2013)[36]	China	Asia	66	8/23	9/26	2-4	6.9 (0.3)	6.7 (0.5)

*CRF*, cooled radiofrequency ablation; *GN, genicular nerve; IA, intra-articular; NA*, not applicable; *PRF*, pulsed radiofrequency ablation; *RFA*, radiofrequency ablation.

**Table 2:** Intervention procedure parameters, results, adverse effects, and limitation of the 12 observational studies included in the current meta-analysis.

First author	Treatment	Treatment	Intervention	Scoring	Follow-up	Results		Adverse effects	Limitation
(year)	target	mode	parameters	methods		Pain	Knee function		
Choi WJ (2011)[7]	GN	RFA	70°C, 90s	NRS, OKS, GPE	1, 3mo	Significantly improved	Significantly improved	None reported	follow-up period was brief; small sample size; lack of data about postprocedure analgesic use.
Shen WS (2017)[10]	IA	RFA	70°C, 120s	VAS, AKSS, SF-36	3mo	Significantly improved	Significantly improved	NA	The technical details of the procedure were unclear; Small sample size; adverse effects were not recorded.
Yuan Y (2016)[16]	IA	PRF	42°C, 6min	VAS, WOMAC	1wk,1,2,3, 6mo	Significantly improved	Significantly improved	Knee joint dropsy were recorded in both groups within 2 weeks after the treatment.	Small sample size; No recording about analgesic consumption.
Gulec E (2017)[17]	IA	PRF	42°C, 10min	VAS,	1wk,1 and 3mo	Significantly improved	Significantly improved	NA	Short follow-up time; adverse effects were not recorded; No recording about analgesic consumption.
				WOMAC					
Rahimzadeh P(2014)[18]	IA	PRF	42°C, 15min	VAS, ROM	2, 4, 12 wk	Moderate improved	Moderate improved	None reported	Short follow-up time; Small sample size
Sari S (2016)[11]	GN	RFA	80°C, 90s	VAS, WOMAC	1, 3mo	Significantly improved	Significantly improved	NA	No recording about analgesic consumption; Short follow-up time
Davis T (2018)[26]	GN	CRF	60°C, 150s	VAS, OKS	1, 3, 6 mo	Significantly improved	Significantly improved	None reported	-
Shen GC (2017)[12]	IA	RFA	70 to 90°C, 3min	VAS,	1, 6mo	Significantly improved	Significantly improved	NA	Small sample size; No recording about analgesic consumption; adverse effects were not recorded.
				Lysholm					
Yang GQ (2015)[13]	GN	RFA	70°C, 120s	VAS, OKS	1, 6, and 12 wk	Significantly improved	Significantly improved	None reported	Small sample size; Short Follow-up time; No recording about analgesic consumption
Yi YS (2012)[14]	GN	RFA	70°C, 120s	VAS, PGA	1, 6, and 12 wk	Significantly improved	NA	None reported	Follow-up period was brief; No recording about analgesic consumption.
Hu Y (2016)[19]	IA	PRF	42°C, 15min	VAS,	1 and 6mo	Significantly improved	Significantly improved	NA	Follow-up period was brief; No recording about analgesic consumption.
				WOMAC					
Yang XL (2013)[36]	GN	RFA	80°C, 180s	VAS	3d, 1 and 2mo	Significantly improved	NA	None reported	follow-up period was brief; small sample size; Lack of scoring method of knee function

*AKSS*, American knee society score; *CRF*, cooled radiofrequency ablation; *GN*, *genicular nerve*; *GPE*, global perceived effect; *IA, intra-articular; NA*, not applicable; *PGA*, patient's global assessment; *PRF*, pulsed radiofrequency ablation; *RFA*, radiofrequency ablation; *ROM*, the knee joint range of motion.

**Table 3:** Subgroup analyses of the WMD of VAS scores between RF group and control group at different time points (one week, one month, and three months).

Subgroups	No. of studies	WMD and 95% CI	Z-value	*P* ^a^	Heterogeneity	
					*I* ^*2*^(%)	*P* ^b^
*One week after treatment*						
Location						
Asia	5	-2.02 (-3.15, -0.89)	3.49	<0.01	92.2	<0.01
Europe	1	-0.65 (-1.22,-0.08)	2.25	0.03	–	–
Blindness						
Yes	2	-0.64 (-1.14,-0.14)	2.52	<0.01	0.0	0.95
No	4	-2.34 (-3.47,–1.22)	4.09	0.01	91.3	<0.01
RF mode						
Pulsed	2	-0.80 (-1.26,-0.34)	3.42	<0.01	0.0	0.36
Radiofrequency ablation	4	-2.25 (-3.43,-1.08)	3.77	<0.01	90.9	<0.01
Concomitant therapy						
Yes	3	-2.77 (-3.71,-1.84)	5.83	<0.01	83.9	<0.01
No	3	-0.77 (-1.19,-0.35)	3.59	<0.01	0.0	0.63
Site of radiofrequency						
Genicular nerve	3	-1.80 (-2.81, -0.78)	3.47	<0.01	74.8	0.02
Intra-articular	3	-1.77 (-3.82, 0.27)	1.70	0.09	97.5	<0.01
Sex ratio (female *vs. *male)						
< 2	2	-1.48 (-3.12, 0.17)	1.76	0.08	92.5	<0.01
≥ 2	4	-1.92 (-3.42, -0.43)	2.52	0.01	94.0	<0.01
*One month after treatment*						
Location						
America	1	-0.90(-1.62, -0.18)	2.46	0.01	-	
Asia	8	-1.42 (-2.10, -0. 73)	4.06	<0.01	89.8	<0.01
Europe	1	-1.86 (-2.87,-0. 85)	3.60	<0.01	-	–
Blindness						
Yes	4	-1.43 (-3. 27, 0.42)	1.51	0.13	93.6	<0.01
No	6	-1.50 (-1.91,–1.08)	7.02	<0.01	67.2	<0.01
RF mode						
Cooled	1	-0.90 (-1.62, -0.18)	2.46	0.01	-	-
Pulsed	5	-0.97 (-1.86,-0.08)	2.13	0.03	84.9	<0.01
Radiofrequency ablation	4	-2.08 (-2.90,-1.25)	4.74	<0.01	80.1	<0.01
Concomitant therapy						
Yes	3	-1.72 (-2.24,-1.21)	6.56	<0.01	48.3	0.15
No	7	-1.34 (-2.20,-0.47)	3.04	<0.01	88.0	<0.01
Site of radiofrequency						
Genicular nerve	5	-1.83 (-2.57, -1.09)	4.82	<0.01	83.0	<0.01
Intra-articular	5	-0.97 (-1.86, -0.08)	2.13	0.03	84.9	<0.01
Sex ratio (female *vs. *male)						
< 2	6	-0.93 (-1.63, -0.23)	2.62	0.01	80.9	<0.01
≥ 2	4	-2.13 (-2.95, -1.31)	5.07	<0.01	78.0	<0.01
*Three months after treatment*						
Location						
America	1	-2.40 (-3.07, -1.73)	7.02	<0.01	-	
Asia	6	-1.17 (-2.39, 0.05)	1.88	0.06	92.2	<0.01
Europe	1	-1.28 (-2.23,-0. 33)	2.65	<0.01	-	–
Blindness						
Yes	4	-0.69 (-2.82,-1.44)	0.63	0.53	94.2	<0.01
No	5	-1.88 (-2.39,-1.37)	7.17	<0.01	53.0	0.08
RF mode						
Cooled	1	-2.40 (-3.07, -1.73)	7.02	<0.01	-	-
Pulsed	4	-0.00 (-1.42,-1.42)	0.00	0.99	88.7	<0.01
Radiofrequency ablation	4	-2.23 (-2.80,-1.67)	7.77	<0.01	39.1	0.18
Concomitant therapy						
Yes	3	-2.01 (-2. 45,-1.57)	8.99	<0.01	0.0	0.99
No	6	-0.98 (-2.45, 0.50)	1.30	0.19	93.3	<0.01
Site of radiofrequency						
Genicular nerve	4	-2.36 (-2.92, -1.80)	8.24	<0.01	35.9	0.20
Intra-articular	5	-0.43 (-1.84, 0.98)	0.59	0.55	92.5	<0.01
Sex ratio (female *vs. *male)						
< 2	5	-0.76 (-2.30, 0.78)	0.96	0.34	93.9	<0.01
≥ 2	4	-2.00 (-2.97, -1.04)	4.07	<0.01	76.3	<0.01

WMD: weighted mean difference; CI: confidence interval.

^a^ *P* value for Z test; ^b^*P* value for Q test.

## Supplementary Materials

Supplementary Materials In order to evaluate knee function improvement among the different RF treatment groups at different observation points, we try to calculate the WMD of WOMAC and OKS. The majority of the results showed that RF treatment did not improve the knee function significantly (see Figures S1 and S2 in supplementary material). Because of the limited studies enrolled in our study, we did sensitivity analysis by excluding studies one by one. The removal of one study each turn showed no significant variation in WMD, which confirmed the stability of our results (see Figure S3 in supplementary material).
