# Supplementary material for: Systematic Review and Meta-Analysis of 12 Randomized Controlled Trials Evaluating the Efficacy of Invasive Radiofrequency Treatment for Knee Pain and Function
Source: Biomed Res Int. 2019 Jun 26;2019:9037510. doi: 10.1155/2019/9037510 (PMC6617930; doi:10.1155/2019/9037510)
Supplement: Supplementary Materials — In order to evaluate knee function improvement among the different RF treatment groups at different observation points, we try to calculate the WMD of WOMAC and OKS. The majority of the results showed that RF treatment did not improve the knee function significantly (see Figures S1 and S2 in supplementary material). Because of the limited studies enrolled in our study, we did sensitivity analysis by excluding studies one by one. The removal of one study each turn showed no significant variation in WMD, which confirmed the stability of our results (see Figure S3 in supplementary material). [file 9037510.f1.pdf]

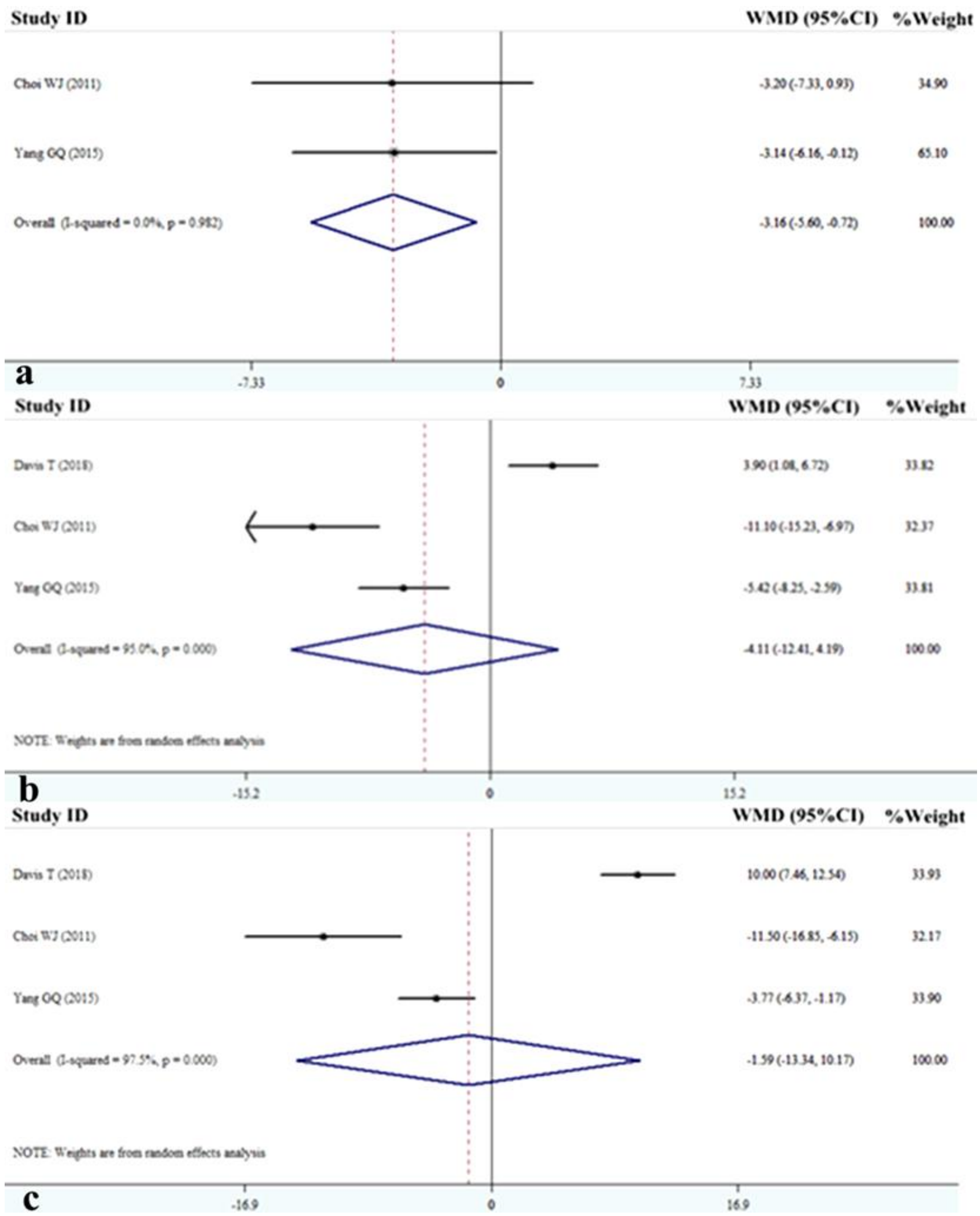

Figure S1. Forest plots for the associations between radiofrequency treatment and OKS scores (a) at 1 week, (b) 1 month, and (c) 3months.

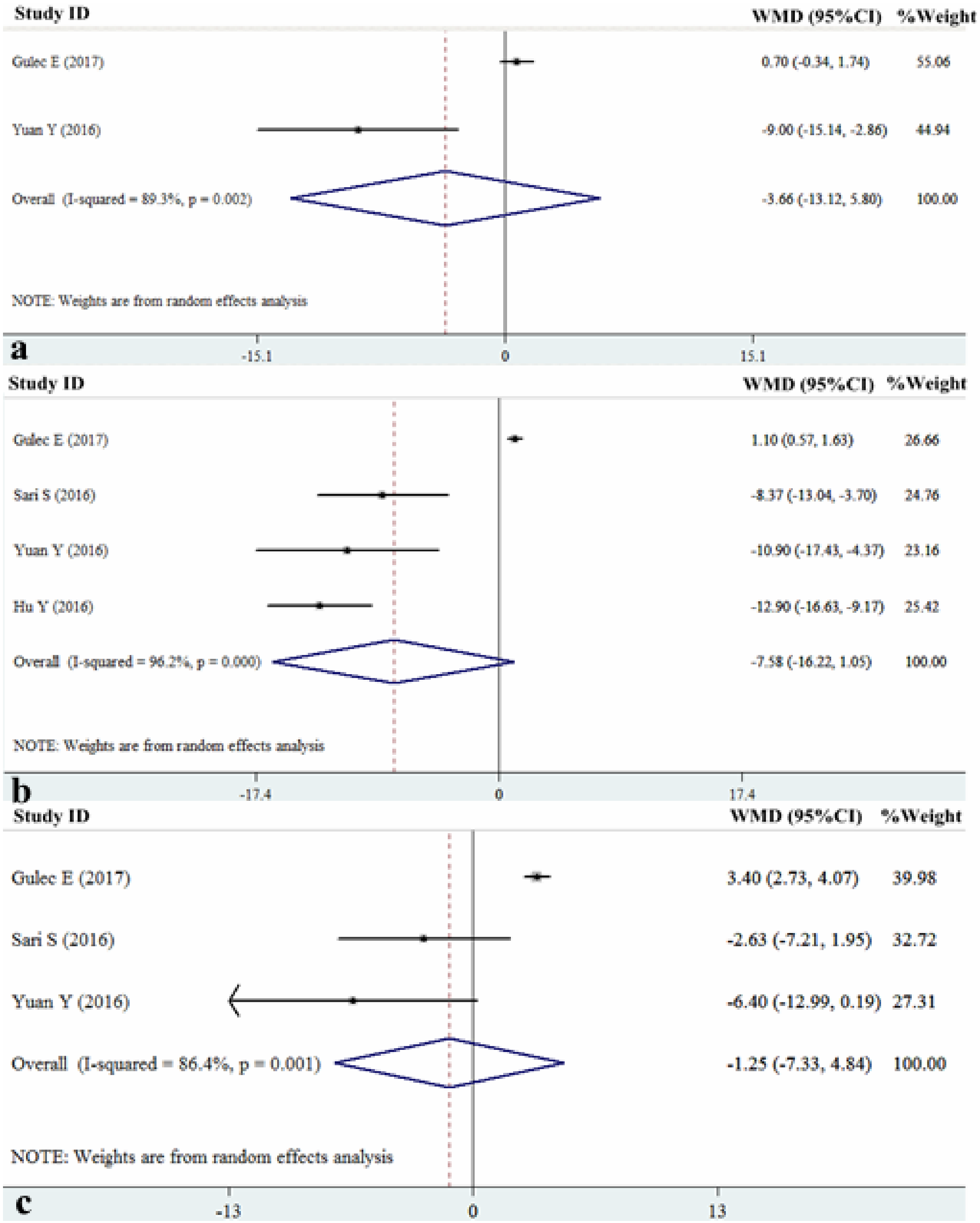

Figure S2. Forest plots for the associations between radiofrequency treatment and WOMAC scores (a) at 1 week, (b) 1 month, and (c) 3months.

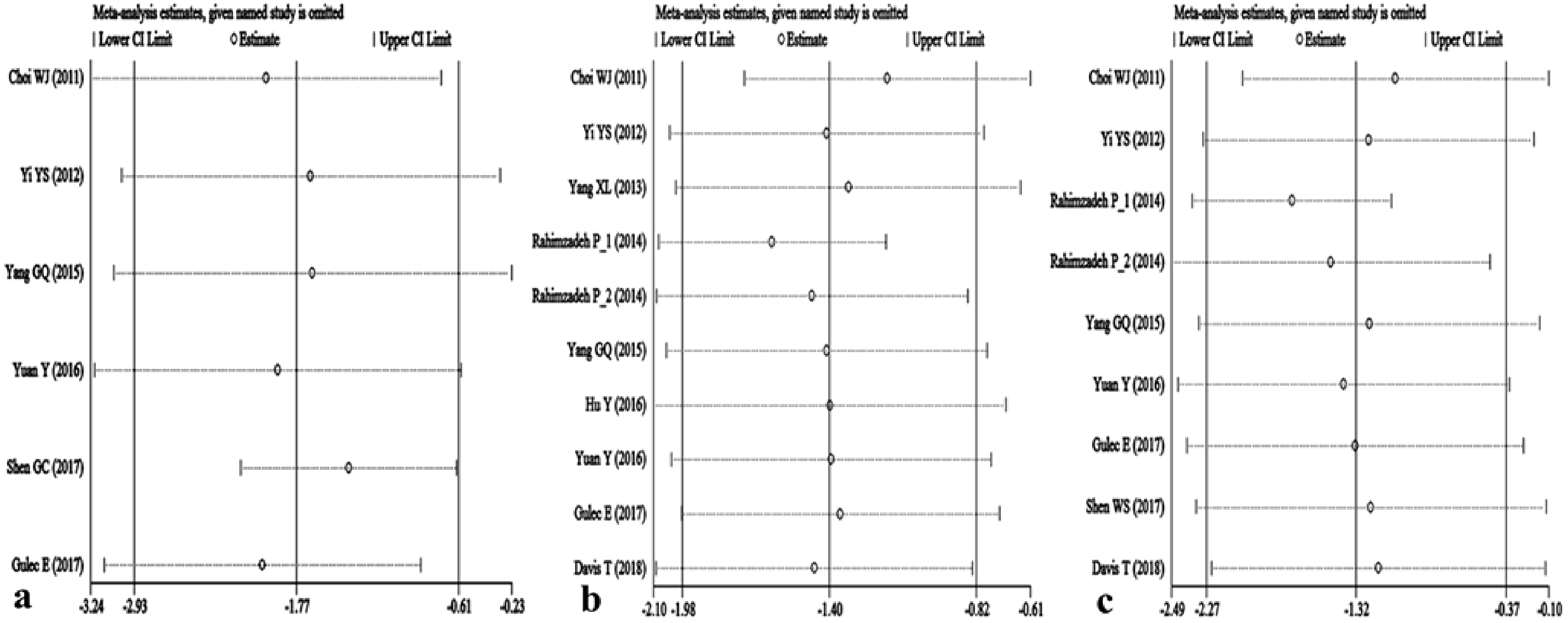

Figure S3. Sensitivity analysis for the associations between radiofrequency treatment and VAS scores (a) at 1 week, (b) 1 month, and (c) 3months.
